# Multiphase progenetic development shaped the brain of flying archosaurs

**DOI:** 10.1038/s41598-019-46959-2

**Published:** 2019-07-25

**Authors:** Vincent Beyrand, Dennis F. A. E. Voeten, Stanislav Bureš, Vincent Fernandez, Jiří Janáček, Daniel Jirák, Oliver Rauhut, Paul Tafforeau

**Affiliations:** 1 European Synchrotron Radiation Facility, 71 Avenue des Martyrs, CS-40220, 38043 Grenoble, France; 2 Department of Zoology and Laboratory of Ornithology, Palacký University, 17.listopadu.50, 77146 Olomouc, Czech Republic; 3 Department of Biomathematics, Institute of Physiology of the Czech Academy of Sciences, Vídeňská 1083, 142 20 Prague 4, Czech Republic; 4 MR Unit, Department of Diagnostic and Interventional Radiology, Institute for Clinical and Experimental Medicine, Vídeňská 1958/9, 142 21 Prague 4, Czech Republic; 5 Institute of Biophysics and Informatics, 1st Medicine Faculty, Charles University, Salmovská 1, 120 00 Prague 2, Czech Republic; 6 Department for Earth and Environmental Sciences and GeoBio-Center, SNSB-Bayerische Staatssammlung für Paläontologie und Geologie, Ludwig-Maximilian-University Munich, Richard-Wagner-Str. 10, 80333 Munich, Germany

**Keywords:** Palaeontology, Imaging techniques

## Abstract

The growing availability of virtual cranial endocasts of extinct and extant vertebrates has fueled the quest for endocranial characters that discriminate between phylogenetic groups and resolve their neural significances. We used geometric morphometrics to compare a phylogenetically and ecologically comprehensive data set of archosaurian endocasts along the deep evolutionary history of modern birds and found that this lineage experienced progressive elevation of encephalisation through several chapters of increased endocranial doming that we demonstrate to result from progenetic developments. Elevated encephalisation associated with progressive size reduction within Maniraptoriformes was secondarily exapted for flight by stem avialans. Within Mesozoic Avialae, endocranial doming increased in at least some Ornithurae, yet remained relatively modest in early Neornithes. During the Paleogene, volant non-neoavian birds retained ancestral levels of endocast doming where a broad neoavian niche diversification experienced heterochronic brain shape radiation, as did non-volant Palaeognathae. We infer comparable developments underlying the establishment of pterosaurian brain shapes.

## Introduction

Among Diapsida, the avian brain is uniquely enlarged relative to body size, which renders birds the only animal group that rivals the encephalisation of mammals^1^. Certain cognitive requirements of vertebrate flight, particularly those demanded in processing sensory input and controlling the intricate flight apparatus^2–5^, have been used to explain important changes in relative volume and organisation of the brain along the evolutionary pathway towards a “flight-adapted” brain^6^. Associated evolutionary patterns, including cranial shape shifts^7–9^ and overall body size reduction along the avian stem^10^, propose paedomorphosis as an important developmental mechanism underlying the establishment of characteristic properties of avian anatomy, including the inflated brain. Such overarching trends in the evolutionary lineage leading up to modern birds are consistent with the hypothesis that heterochronic developments were crucial towards the establishment of archosaurian volancy in general and dinosaurian flight in particular.

Detailed comparative investigations into brain shape and size at the very onset of dinosaurian flight have been incapable of identifying specific endocranial conditions unequivocally linked with dinosaurian volancy. It has been concluded that the brain of the early volant avialan *Archaeopteryx*, considered exemplary for the transition between non-volant and volant theropods^11,12^, did not present an anatomy profoundly contrasting those of non-volant Maniraptoriformes^1^. Only a single cerebral structure, the wulst, until then only recognised in modern birds (Neornithes) but conclusively absent in non-volant Maniraptoriformes and Mesozoic (non-avian) Ornithurae^13,14^, was found to also be potentially present in *Archaeopteryx*^1^. The apparent lack of unambiguous flight-related cerebral adaptations in the oldest volant avialan identified to date has confused our understanding of the relation between inferred dinosaurian volancy and the development of a correspondingly flight-capable brain^15^. Furthermore, because the variation in relative endocranial and cerebral volumes of modern birds and their non-avian ancestors was found to not primarily reflect the presence or absence of powered flight^16^, other geometrical parameters should be explored to resolve the potential influence of volancy on the evolution of the archosaurian brain.

Where early palaeoneurological investigations on lithified cranial fossils typically involved perilous physical casting and occasionally demanded sacrifice of complete osseous braincases, the advent and development of X-ray computed tomography (CT) has enabled reliable and non-destructive reconstruction of the size and shape of an endocranial cavity^17^. Although conventional CT setups typically allow for reliable endocranial visualisation of modern material, fossils encased in and filled with a sedimentary matrix may require the application of more elaborate tomographical techniques, especially when the fossil is preserved in a plate-like slab composed of a lithic substrate with a density comparable to that of the sample of interest. More advanced CT approaches do not only enable the visualisation of samples that cannot be confidently resolved otherwise, but will generally also offer improved contrast and spatial resolution to allow for more reliable reconstruction and comparison of multiple samples, and a better appreciation of deformation incurred during taphonomy. Synchrotron propagation phase-contrast X-ray microtomography (PPC-SRµCT) in particular offers unparalleled contrast and detail in palaeontological data and has emerged as the pre-eminent approach to imaging challenging fossils today^18–23^.

Because brain tissue itself rarely fossilises^24,25^, palaeoneurology resorts to studying the cranial endocast that reflects the surface geometry of the endocranial cavity in which the brain was housed during life^12,17^. Non-avian dinosaurs generally exhibit an overall brain shape resembling the crocodilian brain more strongly than the modern avian brain^26,27^. We aimed to exploit the conservative preservation of the osseous braincase in both extinct and extant taxa while avoiding uncertainty regarding whole-brain volume or exact delimitation of cerebral components. Comparative analysis of endocast geometries covering a wide phylogenetic, size, and shape range confidently permits identification of broadly supported trends relevant to identifying the evolutionary processes that shaped the modern avian brain.

To test the hypothesis that the endocranial cavities of extinct bird-line archosaurs conservatively documented the evolutionary pathway of the brain through time, we compiled a representative database of endocranial shapes encompassing the largest possible diversity of extant and extinct archosaurs, and included selected lepidosaurs as an outgroup. In order to evaluate the influence of developmental heterochrony on the evolutionary trajectory towards modern birds, we furthermore investigated ontogenetic series of extant crocodilians and birds to assess associated brain shape developments while bracketing non-avian dinosaurs phylogenetically^28^. PPC-SRµCT with optimised imaging protocols at beamlines BM05, ID17, and ID19 of the European Synchrotron Radiation Facility (ESRF) ensured reliable yet non-destructive visualisation of the endocasts in two specimens of *Archaeopteryx* and other fossil as well as extant archosaurs^18,29,30^. The data set was supplemented with conventional CT data representing a sufficiently wide selection of modern avian endocasts to resolve endocranial variation across the ecological and body size range occupied by present-day birds. Following qualitative characterisation, shape variation was quantified through two-dimensional landmarking in lateral view and shape changes were subsequently analysed with Principal Component Analysis (PCA). PCA identified dorsal endocast curvature, originating from brain axis flexure and relative telencephalic and cerebellar size, as the dominant shape variable in the data set. The C/D parameter, defined as the ratio of dorsal endocast length following the convex hull between the anteriormost tip of the cerebrum and the opisthion (C) over the linear distance between those two locations (D), was recovered as a straightforward geometric ratio conservatively capturing and quantifying endocast doming. The biological significance of C/D was tested by investigating the relationship between this ratio and physiological properties, including alert head posture^31^ and encephalisation quotient^32^. We compared embryonic C/D development in *Crocodylus niloticus*, *Gallus gallus*, and *Ficedula albicollis* to characterise and compare developmental timing during their *in ovo* trajectories towards revealing heterochronic influences on embryonic ontogeny. We furthermore plotted endocast doming (C/D) against log D for all adult archosaurs subjected to PCA, but also included a broad selection of lepidosaurs, as an outgroup to archosaurs, and an ontogenetic growth series of *Crocodylus niloticus*. This resolved a partially overlapping distribution of archosaurian endocranial shapes and sizes that can be directly compared against the complete ontogenetic variability of *C. niloticus*.

## Results

**Figure 1:**
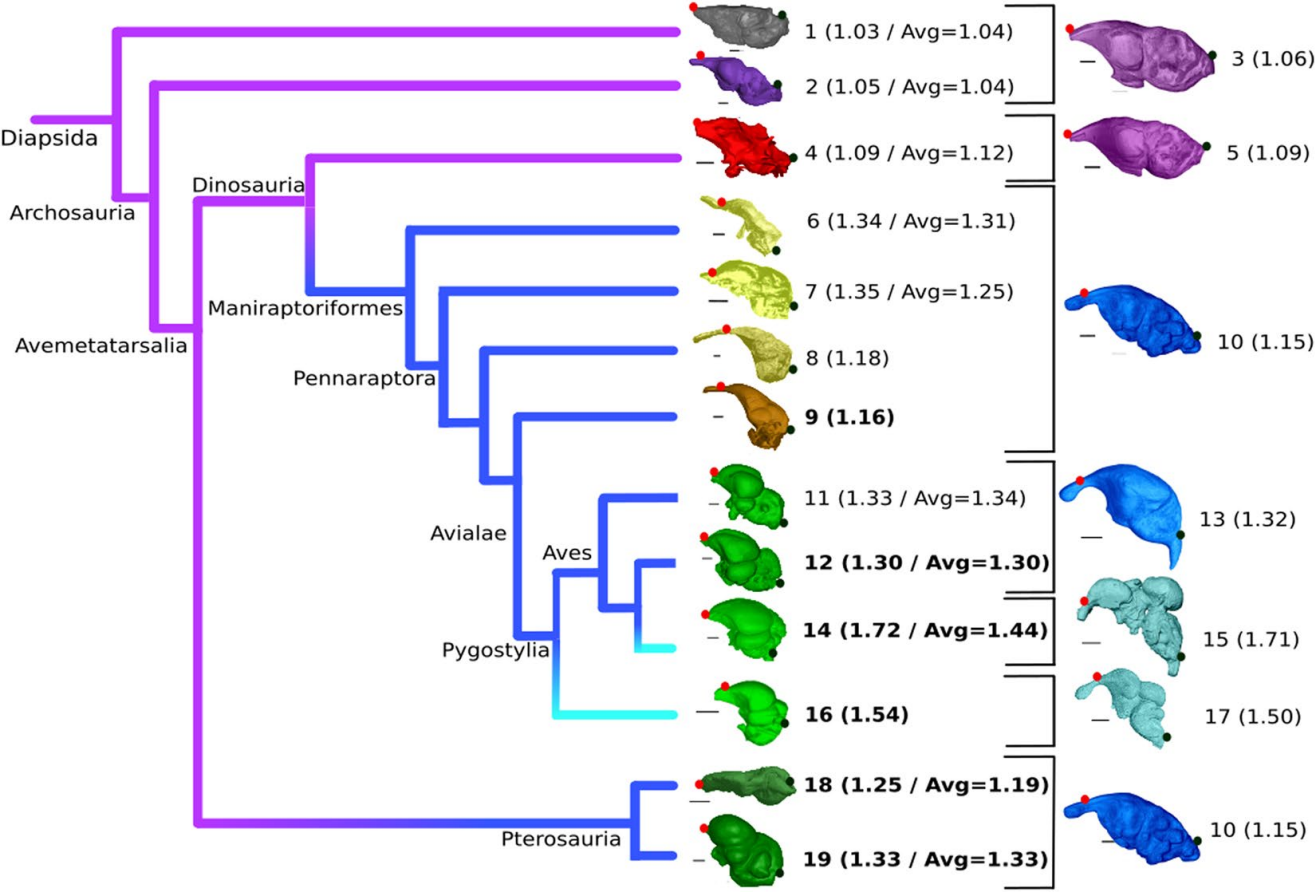
Heterochronic evolution of archosaurian endocranial geometry along the avian stem^29^. Phylogeny of studied archosaurian groups and endocasts of key specimens visualised. Purple lineages exhibit low endocast doming, dark blue lineages exhibit medium endocast doming, light blue lineages exhibit high endocast doming. Volant taxa are indicated in bold, average group doming value (group declared after representative species) and/or measured specific doming value for single-taxon samples provided between brackets. Corresponding crocodilian ontogenetic stage is visualised through the associated endocast most right. Endocasts not to scale, individual scale bar lengths provided in caption. Dots indicate the positions of the anteriormost tip of the cerebrum (red dot) and the opisthion (black dot). Volant taxa are indicated in bold, average group doming value and/or measured specific doming value for single-taxon samples provided between brackets. Taxa presented: *Podarcis muralis* (1; scale bar 0.65 mm; Lepidosauria); *Caiman crocodilus* (2; 5 mm; Crocodylia); One-year-old *Crocodylus niloticus* (3; 2.5 mm); *Arcovenator escotae* (4; 20 mm; Non-maniraptoriform Dinosauria); *C. niloticus* hatchling (5; 2.5 mm); *Struthiomimus altus* (6; 10 mm^36^; Ornithomimosauria); *Incisivosaurus gauthieri* (7; 10 mm^96^; Non-avian Maniraptoriformes); *Halszkaraptor escuillei* (8; 3 mm); *Archaeopteryx lithographica* (9; 2.5 mm); 68-day-old *C. niloticus* embryo (10; 2.5 mm); *Dromaius novaehollandiae* (11; 6 mm; Palaeognathae); *Phasianus colchicus* (12; 3 mm; Galloanserae); 63-day-old *C. niloticus* embryo (13; 3 mm); *Ficedula albicollis* (14; 1.5 mm; Neognathae); 24-day-old *C. niloticus* embryo (15; 1,5 mm); *Cerebavis cenomanica* (16; 5 mm); 41-day-old *C. niloticus* embryo (17; 2.5 mm); *Rhamphorhynchus muensteri* (18; 5 mm^97^; Rhamphorhynchidae); *Araripesaurus santanae* (19; 4.5 mm; Azhdarchidae).

**Figure 2:**
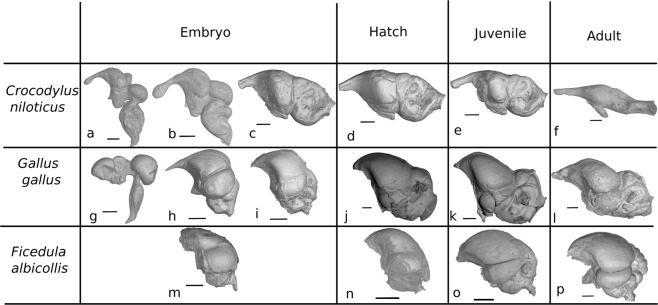
Endocranial shape changes during ontogeny of crocodilian and avian taxa. (**a**) 29-day-old embryo of *Crocodylus niloticus* (scale: 1.5 mm); (**b**) 41-day-old embryo of *C. niloticus* (scale: 2.5 mm); (**c**) 93-day-old embryo of *C. niloticus* (scale: 3 mm); (**d**) Hatchling of *C. niloticus* (scale: 3 mm); (**e**) One-year-old juvenile of *C. niloticus* (scale: 4 mm); (**f**) Adult of *C. niloticus* (scale: 15 mm); (**g**) 5.5-day-old embryo of *Gallus gallus* (scale: 1.5 mm); (**h**) 12-day-old embryo of *G. gallus* (scale: 3 mm); (**i**) 19-day-old embryo of *G. gallus* (scale: 3.5 mm); (**j**) Three-week-old juvenile of *G. gallus* (scale: 2 mm); (**k**) Six-week-old juvenile of *G. gallus* (scale: 3 mm); (**l**) Adult of *G. gallus* (scale: 3.5 mm); (**m**) Six-day-old embryo of *Ficedula albicollis* (scale: 1.5 mm); (**n**) Hatchling of *F. albicollis* (scale: 2.5 mm); (**o**) Juvenile of *F. albicollis* (scale: 2.5 mm); (**p**) Adult of *F. albicollis* (scale: 2 mm). Stages a and g represent the brain shape at the end of the first third of *in ovo* development, stages b, h and m represent half of the *in ovo* development, stages c and i represent the final *in ovo* condition before hatchling.

**Figure 3:**
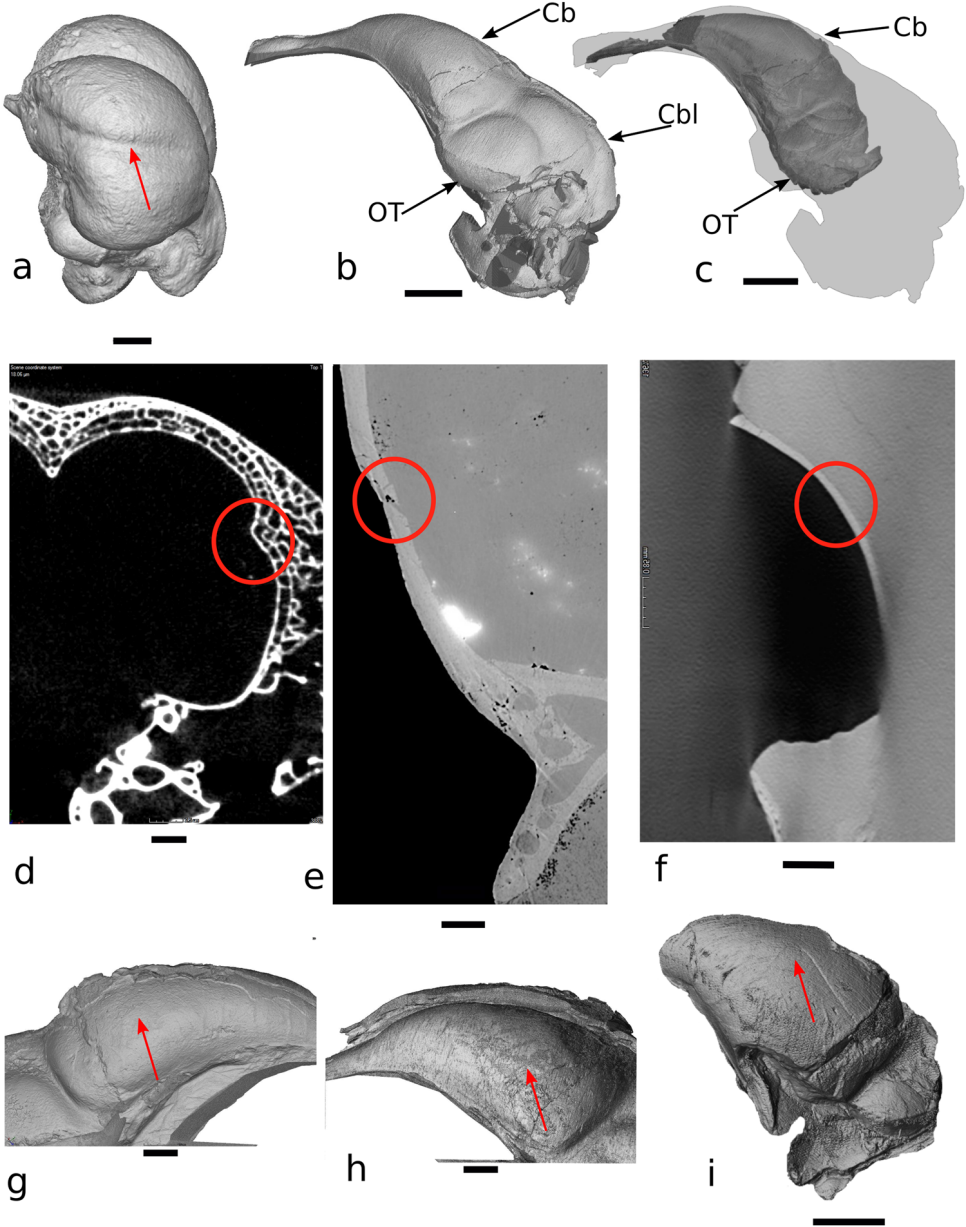
Wulst expression in a modern avian endocast compared with the condition of *Archaeopteryx*. (**a**) Three-dimensional visualisation of the endocast of the snowy owl *Bubo scandiacus*; (**b**) Left lateral view of the complete endocast of the London specimen of *Archaeopteryx*; (**c**) Reconstructed partial endocast of the Munich specimen of *Archaeopteryx* superimposed on grey endocast silhouette of the London specimen of *Archaeopteryx*; (**d**) Slice view of the braincase of *B. scandiacus* at position of osseous delimitation of wulst (in red circle); (**e**) Slice view through the braincase of the London specimen of *Archaeopteryx* at position of delimitation of potential “wulst^1^” presenting taphonomic fracture (in red circle); (**f**) Slice view of the braincase of the Munich specimen of *Archaeopteryx* at position homologous to the delimitation of potential “wulst^1^”; (**g**) Detailed right lateral view of the endocast of the London specimen of *Archaeopteryx*; (**h**) Detailed left lateral view of the endocast of the London specimen of *Archaeopteryx*; (**i**) Left lateral left view of the telencephalic region of the Munich specimen of *Archaeopteryx* endocast; the red arrows indicate the position of the wulst on the surface of the *B*. *scandiacus* endocast and proposed wulst locations on the surface of the London and Munich specimens of *Archaeopteryx*. Abbreviations: Cb-Cerebrum; Cbl-Cerebellum; OT-Optic tectum. Scale bar: a-4 mm; b-c-3.5 mm; d-0.25 mm; e-0.3 mm; f-0.85 mm; g-h-i-1.5 mm.

The endocranial cavity of archosaurs exhibits one of two general shapes most strongly expressed in lateral view. The first generalised endocast geometry is principally elongated and does not exhibit clear delimitation of cerebral structures (Fig. 1). This condition is observed in modern crocodiles (Fig. 2) and most non-maniraptoriform dinosaurs, and is importantly shared with lepidosaurs (Fig. 1). Pterosaurs show a highly bulbous and very well defined endocranial anatomy (Fig. 1). Although non-avian Maniraptoriformes exhibit some variability in endocranial shape and cerebral border delimitation (Fig. 1), their endocasts are generally quite bulbous and cerebral compartmentalisation is at least subtly expressed on the endocast surface. Modern birds (Neornithes) present a very bulbous braincase endocast that features a very comprehensive delimitation of cerebral structures (Figs 1 and 2). Birds furthermore developed a unique cerebral attribute located on the top of the telencephalon that is absent in all other archosaurian lineages and is known as the wulst^1^ (Fig. 3).

Although a wulst has been proposed to potentially be present in the London specimen of the stem avialan *Archaeopteryx*^1^, we found it is conclusively absent in the two *Archaeopteryx* specimens evaluated here (Fig. 3)^14^. Where the “cerebral indentation” resolved on conventional tomographical data of the London Specimen was proposed to potentially indicate the presence of a wulst homologous to those of Neornithes^1^, high-quality PPC-SRμCT data from the same specimen demonstrates that this feature actually resulted from partial collapse of the braincase roof during taphonomy. The Munich Specimen preserves the equivalent section of the osseous braincase in superb detail but presents a smooth and continuous internal topography that also lacks anatomical indications for the presence of a wulst on the brain or a wulst-like projection on the cerebral endocast of *Archaeopteryx* (Fig. 3).

**Figure 4:**
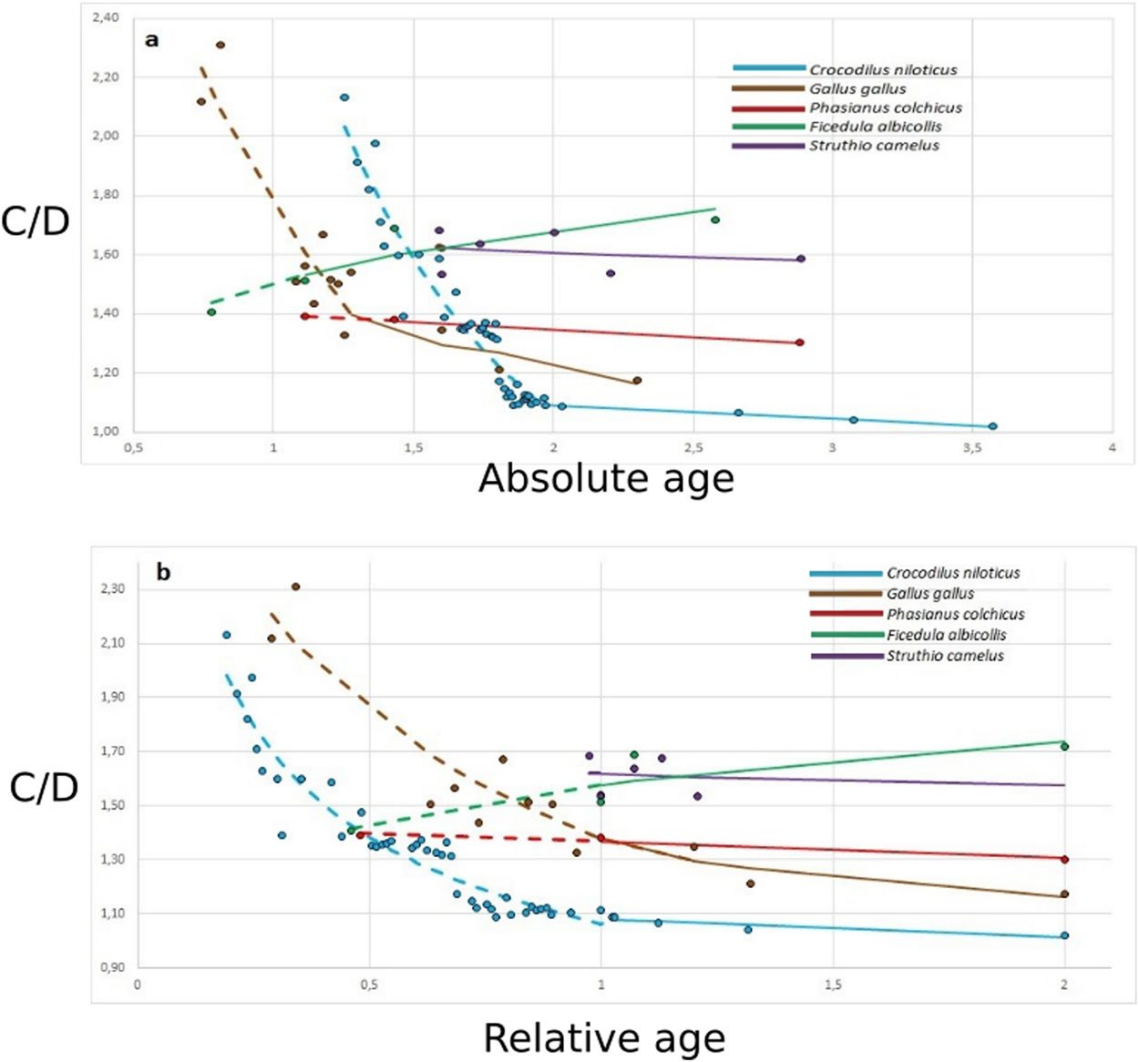
Bivariate plots of developmental age versus endocranial doming (C/D) for selected archosaurian taxa. Ages presented as log-transformed age in days (**a**) and as developmental-stage-normalised ages relative to hatchling (1; **b**). Developmental series are divided in pre-hatchling (dashed line) and post-hatchling (continuous line) stages. Markers most right reflect the condition at sexual maturity.

In archosaurs, embryonic endocranial shape changes can only be reliably visualised in extant taxa, as poor cranial ossification during early development renders representative ontogenetic series of fossil archosaurs *in ovo* insufficiently available. During *in ovo* embryonic development of the crocodile *Crocodylus niloticus*, progressive endocranial elongation is associated with a decreasing expression of cerebral structures on the endocast surface (Fig. 2). Straightening of the brain continues post-hatching but does so more gradually and to a markedly lesser degree than during embryonic development. Consequently, shape variation is substantially larger in embryonic phases than across post-hatching ontogeny. During embryonic development of *C. niloticus*, the long axis of the braincase endocast rotates anterodorsally from a nearly vertical orientation during the first embryonic trimester to the horizontal configuration that characterises hatchlings and adults alike (Fig. 2). The neornithine ontogenetic pathways expressed in endocast shape contrast those of crocodilians. During *in ovo* embryonic development of the galliform *Gallus gallus*, only partial endocast straightening occurs while the clear expression of cerebral compartmentalisation persists. Endocast straightening halts during *in ovo* development of *G*. *gallus* and remains constant during the subsequent ontogenetic stages (Fig. 2). Developmental endocast shape shifts in the more restrictively sampled paleognath *Struthio camelus* (n = 7) and galliform *Phasianus colchicus* (n = 3) appear to follow the same pattern (Fig. 4). More derived (neoavian) birds, such as the passeriform *Ficedula albicollis*, also conserve embryonic levels of cerebral compartmentalisation throughout life. However, their adult braincase endocast appears to be even more domed, corresponding to a more coiled brain, than those of six-day-old embryonic individuals (Fig. 4). As such, qualitative principal shape change of the braincase endocast during ontogeny in *F. albicollis* after the sixth day of embryonic development seems to be reversed to that observed for *C. niloticus* (Fig. 2).

**Figure 5:**
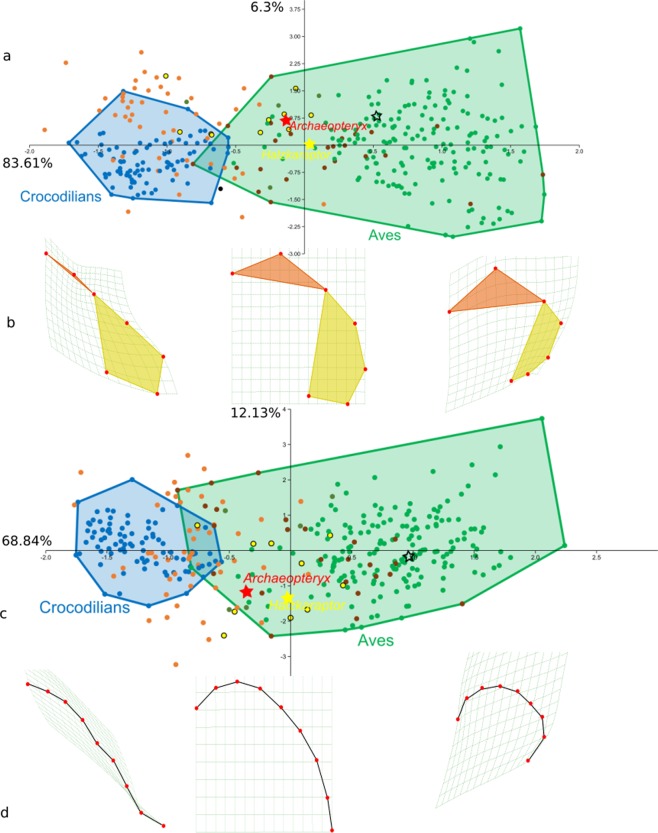
PCA plots for Type I landmarks and semilandmarks, and associated dominant dorsal endocast shape changes. (**a**) Principal Component Analysis plot for Type I landmarks; (**b**) Summary of endocranial shape change along PC1 for Type I landmarks; dorsal endocast contours on deformation grid from average; (**c**) Principal Component Analysis plot for semilandmarks; (**d**) Summary of endocranial shape change along PC1 for semilandmarks; dorsal endocast contours on deformation grid from average. Crocodilian specimens are indicated by blue dots, the crocodilian distribution is delimited by a blue hull. Non-maniraptoriform dinosaurs are indicated with orange dots, Maniraptoriformes with yellow dots, Paleognathae with brown dots, and Neognathae with green dots. The avian distribution (Paleognathae + Neognathae) is delimited by a green hull. Endocast shape variations (**b**,**d**) include landmark positions as red dots. Coloured hulls (in **b**) delimit cerebral domains occupied by the telencephalon (orange) and rhombencephalon (yellow).

The qualitative patterns in endocranial shape variation across (sub-) adult archosaurs are corroborated by Principal Component Analysis. PCA morphospaces for two-dimensional Type I landmarks and semilandmarks assigned to archosaurian endocasts in lateral view (see Methods) exhibit broadly comparable shape distributions (Fig. 5a,c) that are strongly correlated (93%, Extended Data Fig. 1a). For both landmark types, PC1 explains the majority of shape variation (80% for Type I landmarks and 89% for semilandmarks, Extended Data Fig. 1b,c). In both Type I and semilandmark morphospace, PC1 primarily captures whole-endocast shape changes in a pattern transforming a virtually straight dorsal endocast margin to a strongly dorsally domed endocast (Fig. 5b,d), consistent with visual observations previously described. This pattern arises through an overlapping succession of phylogenetic clusters representing, in order of increasing PC1 scores, crocodilians, non-maniraptoriform dinosaurs, non-avian maniraptoriforms, and Neornithes. In Type I landmark morphospace, PC1 additionally captures endocast shape shifts that reflect intra-endocast shape variability in the cerebral domains roughly corresponding with the telencephalon and the rhombencephalon (Fig. 5b). Although a relative progressive increase in telencephalic size appears to typically accompany increasing PC1 scores, the referred pattern involves several non-homologous morphological transformations and thereby fails to capture truly comparable shape shifts across the phylogenetically diverse archosaurian assemblage. This renders the semilandmark set most appropriate for visualising and studying whole-endocast shape variation in the data set studied here.

The dominant shape shift in the semilandmark set, reflected in PC1, can be reliably expressed as a ratio dividing the total distance of the dorsal margin of the endocast between the anteriormost point of the telencephalon and the dorsalmost point of the foramen magnum (C) over the shortest distance between these two points (D). The correlation between PC1 in semilandmark PCA and the novel geometrical ratio C/D was found to be substantial (88%, Extended Data Table 1), which reinforces employment of this ratio as a straightforward yet robust measure of the dominant endocast shape variability in our data set.

The doming ratio C/D does not correlate with body mass (R^2^ = 0.15; Kendall = −0.28, Extended Data Fig. 4, Extended Data Table 4). C/D also correlates poorly with the orientation of the lateral semicircular canal^31^ (R^2^ = 0.01; Kendall = −0.05, Extended Data Fig. 4, Extended Data Table 4), suggesting that C/D does not relate to (alert) head posture.

Endocast doming and encephalisation quotient^32^ were found to correlate significantly across the complete archosaurian set (R2 = 0.38; Kendall = 0.48, Extended Data Fig. 4, Extended Data Table 5), suggesting that doming may be employed as a proxy for EQ. This correlation increases when tested for exclusively non-avian archosaurs (R^2^ = 0.7983, Kendall = 0.67, Extended Data Fig. 4, Extended Data Table 5) because endocast doming and EQ are not significantly correlated within modern birds (R^2^ = 0.136, Kendall = −0.11 in Paleognathae/Galloanserae, and R^2^ = 0.09; Kendall = 0.24 in Neoaves; Extended Data Fig. 4, Extended Data Table 5).

**Figure 6:**
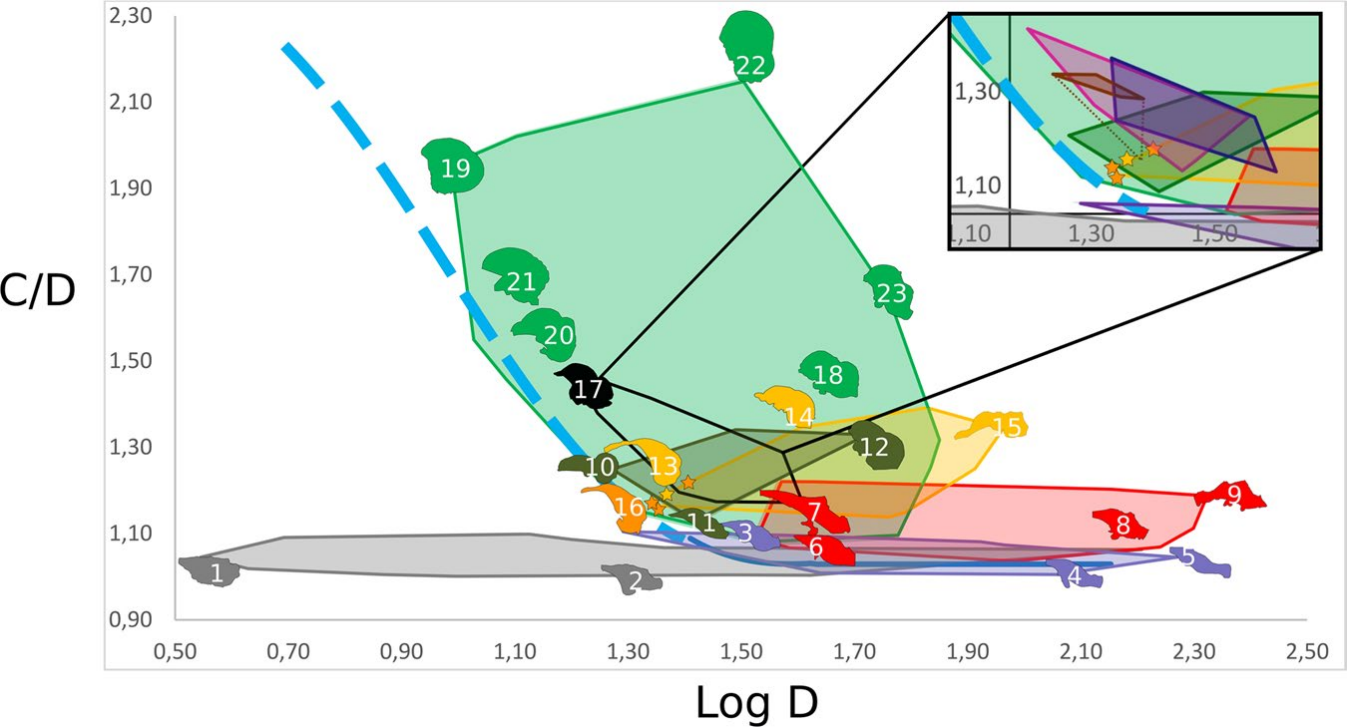
Bivariate plot of endocranial doming (C/D) versus log-transformed endocast length (Log D). Blue indicates crocodilian pre-hatchling (dashed light blue line) and post-hatchling (continuous dark blue line) ontogenetic trajectories. Coloured hulls delimit diapsid groups: Lepidosauria (grey), non-maniraptoriform Dinosauria (red), non-avian Maniraptoriformes (yellow), Pterosauria (dark green), volant non-neoavian birds (black), and non-volant Paleognathae and Neoaves (light green). The inset reflects the endocranial diversity of extant volant non-neoavian taxa: volant Paleognathae (brown), Anseriformes (dark blue), and Galliformes (dark pink). Dashed brown line visualises the addition of the extinct volant paleognath *Lithornis plebius* to extant flying Paleognathae. Visualised endocasts mark the positions of individual specimens: 1-*Podarcis muralis*; 2-*Varanus exanthematicus*; 3-*Caiman crocodylus*; 4-*Crocodylus niloticus*; 5-*Alligator mississipiensis*; 6-*Heterodontosaurus tucki*; 7-*Psittacosaurus lujiatunensis*; 8-*Arcovenator escotae*; 9-*Tyrannosaurus rex*; 10-*Rhamphorhynchus muensteri*; 11-*Paraspicephalus purdoni*; 12-*Araripesaurus santanae*; 13-*Halszkaraptor escuillei* (yellow star); 14*-Incisivosaurus gauthieri*; 15-*Struthiomimus altus*; 16-*Archaeopteryx lithographica* (orange stars); 17-*Phasianus colchicus*; 18-*Leptoptilos crumeniferus*; 19-*Thalurania furcata*; 20-*Cerebavis cenomanica*; 21-*Ficedula albicollis*; 22-*Strix nebulosa*; 23-*Struthio camelus*.

Across the entire size range, adult lepidosaurs (C/D range 1.00–1.16) and crocodilians (C/D range 1.01–1.10) exhibit strongly elongated endocast shapes (average C/D = 1.04) that broadly overlap in morphospace (Fig. 6, Extended Data Fig. 2). Non-maniraptoriform dinosaurs (C/D range 1.04–1.30) are contained within a hull that marginally overlaps the shape ranges of lepidosaurs and crocodilians but generally reflects distinctly more domed endocasts (average C/D = 1.12, difference from the ancestral shape + 0.08). The hull for non-avian maniraptoriforms (C/D range 1.09–1.54, average C/D = 1.25, +0.21) partially overlaps the upper range of endocast doming in non-maniraptoriforms but does not overlap the lepidosaurian or crocodilian hulls. Endocast curvature measures of this group furthermore largely agree with those recovered for pterosaurs (C/D range 1.13–1.34, average C/D = 1.25, +0.21). Notably, the basal pterosaur *Rhamphorhynchus muensteri* already exhibits a C/D of 1.24. Volant palaeognathous birds, including the Paleocene-Eocene genus *Lithornis* (C/D range 1.20–1.38, average C/D = 1.33, +0.29), share a discrete domain in endocast shape and size morphospace with Galliformes (C/D range 1.17–1.48, average C/D = 1.30, +0.26) and Anseriformes (C/D range 1.17–1.41, average C/D = 1.29, +0.25) that partially overlaps with the hulls of non-avian maniraptoriforms and pterosaurs. Sparsely available metrical and endocast data on a cranium of the Eocene anseriform *Presbyornis* sp. (USNM 299846)^33,34^ indicates a C/D ratio between 1.24 and 1.37 for this taxon, which is well within the range occupied by present-day anseriforms. Non-volant palaeognaths (“ratites”) share their average degree of endocast doming with volant palaeognaths but encompass a relatively broad C/D range (C/D range 1.10–1.71, average C/D = 1.34, +0.30). The neoavian radiation spans a particularly broad endocast shape variability (C/D range 1.08–2.15, average C/D = 1.49, +0.45) that partially overlaps with all other groups but also includes the highest C/D values recorded. Notably, the Late Cretaceous ornithurine *Cerebavis cenomanica* exhibits a C/D ratio in the range of modern volant neoavians (C/D = 1.54), which is higher than in any volant palaeognath, galliform, or anseriform.

The non-volant dromaeosaur *Halszkaraptor* and the early volant avialan *Archaeopteryx* present comparable endocranial geometries that are intermediate between those of non-maniraptoriform dinosaurs and those found in modern birds^6^ (Fig. 6). *Halszkaraptor* exhibits a C/D of 1.18, and precise measurements on the London specimen of *Archaeopteryx* and conservative estimates obtained for the Munich specimen of *Archaeopteryx* through its partially preserved left telencephalic hemisphere yielded C/D values of 1.16 and 1.17, respectively.

During *in ovo* embryonic development of *Crocodylus niloticus*, C/D values decrease from 2.13 on day 18 to (on average) 1.11 on day 93 (Fig. 4). Although post-embryonic maturation does involve a certain degree of brain straightening^32^, post-hatching endocast doming levels are always low in *C. niloticus*. *C. niloticus* was found to exhibit C/D values upon hatching, thirteen days after hatching, and in the first and third year of life, of 1.09, 1.09, 1.06, and 1.04, respectively. Such values, up to C/D = 1.10, were encountered in the adult crocodilian set as well. Comparison of ontogenetic C/D development between *Crocodylus niloticus* and *Gallus gallus* from early *in ovo* embryonic stages to adulthood revealed shape trajectories with a statistically significant divergence (T value = 2.61, p = 0.0057, Extended Data Table 2). Different than in *C. niloticus* and *G. gallus*, ontogenetic C/D values obtained for *Ficedula albicollis* describe an *in ovo* shape trajectory including a stage during which C/D increases.

## Discussion

Crocodilians and birds, the only archosaurian groups with extant representatives, present morphological end members of adult archosaurian brain shape variation. The linear “crocodilian” brain, exhibiting sequential arrangement of neuroanatomical domains and a caudally positioned connection to the spinal column, accounts for the primitive archosaurian condition^35^ that is shared with the archosaurian outgroup Lepidosauria^23,36,37^ (Fig. 6). In contrast, the avian brain and, to a lesser extent, the pterosaurian brain exhibit a strongly domed geometry that results from a relatively inflated cerebrum, more complex spatial distribution of the neuroanatomical domains following stronger brain axis flexure, and a more ventrally positioned connection to the spinal column^1^ (Figs 1 and 6).

The absence of a wulst in *Archaeopteryx* restricts its confirmed distribution to modern and a few fossil Cenozoic Neornithes^38,39^. Nevertheless, the occurrence of the wulst in both *Struthio* and *Passer* indicates that it was already present in the last common neornithine ancestor, effectively rendering the wulst a unique synapomorphy of an ornithuromorphan clade that excludes *Cerebavis*^14^ but includes all modern birds. This contrasts the suggested convergent apparition of a wulst in archaeopterygian and neornithine ancestors^1^. As the wulst is known to serve binocular vision^40^, navigation during migration^41^, and various other somatosensory functions^42^, it may have contributed to neornithine survival of the K/Pg crisis, in which all other dinosaurs perished, and facilitated the rapid Cenozoic radiation of the clade.

In association with progressive size reduction of bird-line dinosaurs^10^, analyses of cranial shape trajectories across archosaurian phylogeny have revealed relatively short ontogenetic trajectories in eumaniraptorans that are consistent with paedomorphosis by progenesis (where accelerated somatic maturation results in a truncated ontogeny relative to ancestral taxa) and explain the relative brain enlargement in Eumaniraptora as a paedomorphic feature^7,43–45^. Progenesis has been argued to represent the most important heterochronic pathway culminating in evolutionary novelties^46,47^. Progressive progenetic expression recognised in the evolutionary history of bird-line dinosaurs can be mirrored on corresponding (and increasingly earlier) developmental stages in the crocodilian ontogenetic series (Fig. 1). Furthermore, the retained posteroventral rotation of the adult avian brain relative to those of crocodilians^35^ and non-avian dinosaurs^48^, such as *Archaeopteryx*^11^ (Fig. 3), contributed importantly to the collapse of the facial region in birds^7^.

In crocodiles, ontogenetic progression has been demonstrated to involve an “unrollment” of the brain through a reduction of cephalic and pontine flexures and relative increase in cerebellar volume, olfactory tract length, and olfactory bulb size^35^. This is readily recognisable in endocast geometry as the embryo develops *in ovo* (Fig. 1). Comparison of endocranial shape through (early) ontogeny in *Crocodylus niloticus* and *Gallus gallus* (Fig. 4a) illustrates disparate C/D pathways most strongly diverging during embryonic development. Both *C. niloticus* and *G. gallus* hatchlings immediately leave the egg without obligate parental assistance^49–52^. Despite different incubation periods (circa 90 days for *Crocodylus niloticus*^53^ and circa 21 days for *Gallus gallus*^54^), this indicates that hatchlings of both taxa represent equivalent developmental stages upon hatchling, which is corroborated by the observation that near-adult C/D values are observed in the hatchlings of each species. *In ovo* embryonic reduction of C/D in *G. gallus* proceeds more rapidly than in *C. niloticus* in absolute time (Fig. 4a) but occurs decelerated relative to *C. niloticus* when both trajectories are normalised to the same developmental stage (Fig. 4b). In effect, *in ovo* decrease of C/D virtually halts upon hatching when (nearly) adult C/D ratios are achieved in *G. gallus* whereas embryonic C/D decrease in *C. niloticus* proceeds below adult ratios of *G. gallus* towards values approaching those of the adult crocodilian condition upon hatching. Consistent with the progenetic signature recognised in overall cranial shape^7^, the divergence between *C. niloticus* and *G. gallus* in magnitude and timing of endocranial shape shifts during embryonic development is best explained by progenesis in bird-line archosaurs. This interpretation is reinforced by the reduced olfactory tracts and bulbs, a relatively smaller cerebellum, and posteroventral rotation of the adult avian brain relative to the crocodilian condition, as these characters are invariably shared with embryonic crocodilians but not with later crocodilian ontogenetic stages.

*Halszkaraptor* exhibits a high level of endocast coiling among non-avian maniraptoriform dinosaurs (Fig. 3), as does the small dromaeosaurid maniraptoriform *Bambiraptor feinbergi*^55^ (C/D = 1.15). Despite the clearly non-volant anatomy of Halszkaraptorinae and its ancestors^29^, *Halszkaraptor* does combine a body size within the range of flying birds with a degree of endocast coiling shared with some modern birds. *Archaeopteryx* also shows endocast coiling within the range of Maniraptoriformes. The endocranial geometry of *Archaeopteryx* is broadly shared with small yet non-volant maniraptorans, particularly *Halszkaraptor*, indicating that its endocranial shape resulted mainly from heterochronic developments at the root of Paraves and did not arise in the context of its incipient dinosaurian volancy. Progenetic reduction in the relative size of the adult olfactory bulbs, as observed in modern birds, was not yet achieved in *Archaeopteryx*^11^, which has been used to infer an olfactory acuity ranging from “typical to high” in *Archaeopteryx* relative to Neornithes^56^. This again illustrates that particular properties of the characteristic avian endocranial anatomy arose within already volant Avialae, implying they did not represent absolute functional prerequisites for flight but rather adaptations towards improved flight or other ecological demands.

The Mesozoic ornithurine *Cerebavis*^57^ is only known from an incomplete and abraded skull preserving a sufficiently complete braincase to reliably reveal a brain shape involving strong flexure and a “stacked” geometry, similar to those of some extant Neornithes^14^. The light cranium itself was furthermore reported to exhibit a degree of fusion indicating advanced flight adaptation, such as also seen in Neoaves^14^.

The current avian phylogeny^58^, coupled with the recent recognition of a basal anseriform clade that originated before the end-Cretaceous extinction event^59^, indicates that the divergences between Palaeognathae-Neognathae, Galloanserae-Neoaves, and Galliformes-Anseriformes all occurred during the Cretaceous^60^. This implies that at least four extant avian clades (Palaeognathae, Galliformes, Anseriformes, and Neoaves) crossed the K-Pg boundary and subsequently negotiated an obligatory non-arboreal phase during the earliest Paleogene^61^. All living birds, including flightless palaeognaths, descended from volant stock^62^, although likely not from dexterous flyers comparable to aerial specialists within modern Neoaves. Furthermore, the present-day palaeognathous diversity originated exclusively during the Paleogene^58^. This group stemmed from a volant tinamou-like ancestor akin to the earliest Paleogene (and possible latest Mesozoic)^63^ members of the clade Lithornithidae^64,65^, despite the true (but presently obscured) origin of Palaeognathae possibly having occurred as far back as the Early Cretaceous^65^.

Within Aves, the galliforms, anseriforms, and volant palaeognaths occupy discrete, restricted, yet broadly overlapping hulls in C/D vs log D morphospace (Fig. 6). This particular domain is also shared with small non-avialan Maniraptoriformes and *Archaeopteryx*, which indicates that these groups exhibit generally similar endocast doming levels at comparable endocast lengths. All referred taxa share essentially ground-dwelling ecologies^66^, which, for the referred avians, likely aided their survival of the K-Pg event (possibly in broadly “shorebird-like” ecologies)^67^ and were retained after the earliest Paleogene recovery of arboreal habitats^61^. The overall C/D range shared by modern-day members of basal avian sister taxa that have occupied analogous habitats since the K-Pg event suggests their level of endocast doming preserved the ancestral, Mesozoic condition for Neornithes. This conclusion is supported by the pivotal positions of *Lithornis* and *Presbyornis* in C/D versus log D morphospace (C/D of 1.20 for *Lithornis* and 1.23 for *Presbyornis*; see Extended Data Table 1) close to those of tinamous and modern anseriforms, respectively, which were all recovered in relative proximity to those of *Archaeopteryx*. Notably, volant archosaurs that succumbed to extinction in the K-Pg event, such as non-avian members of Ornithurae (Ornithurae *sensu*^68^; e.g. *Cerebavis*), the Enantiornithes, and the Pterosauria, may have exhibited endocast coiling levels exceeding those of non-neoavian birds. Nevertheless, their physiological and ecological demands proved incompatible with the global short- and long-term effects of the Chicxulub impact event^61^.

As such, the proportionally large endocast size and C/D range of secondarily non-volant extant Palaeognathae reflects the Paleogene radiative colonisation of ecological niches by volant ancestors followed by independent evolutionary histories after these taxa became flightless^69^.

Most striking is the large diversity in C/D across a broad range of endocast sizes seen in Neoaves that dominates the C/D vs log D plot (Fig. 6). Although Neoaves originated in the Mesozoic^58,61,70^, no Mesozoic neoavians are presently known. The highly diverse and speciose distribution of present-day Neoaves arose during and following their explosive early Paleogene ecological radiation^61,67^. In a comparative context, we conclude that the degree of endocast doming in *Cerebavis* exceeding those of volant non-neoavians was achieved independently from the (Paleogene) neoavian radiation. This observation is consistent with an expression of cognitive challenges surpassing those of the largely ground-dwelling ecologies retained by non-neoavian birds included in our study, and may indicate progressive adaptation to more demanding aerial requirements in *Cerebavis*. Similar progenetic developments appear to have coincided with the origins of other volant archosaurian clades, such as earlier at the root of Pygostylia, but also in Pterosauria (Fig. 1). Progenesis was involved in the establishment of anatomical prerequisites for archosaurian volancy on more than one occasion, albeit through exaptation of features that arose in a non-volant context. Secondary complexification of flying habits within these taxa may have driven progressive reorganisation, such as reflected in the broad neoavian endocranial diversity. C/D ratios obtained for the passerine *Ficedula albicollis* during *in ovo* development (Fig. 4) suggest a substantially different *in ovo* C/D pathway characterised by a chapter of C/D increase rather than continuous decrease towards hatchling, as described in crocodiles and basal Neoaves. This indicates a developmental strategy (partially) uncoupled from the generalised non-neoavian archosaurian C/D curve and may have originated to meet the cognitive requirements required for elaborate flight in ecological niches adopted by (passeriform) Neoaves. Nevertheless, Mesozoic chapters of basal avialan and, later, ornithurine brain inflation did not involve the development of a wulst of wulst-like homologue. Until a wulst is positively identified in a (Mesozoic) ornithurine closer to Neornithes than *Cerebavis*, the wulst is again considered a uniquely neornithine synapomorphy^14,17^.

## Conclusions

Among extant birds, volant palaeognaths, galliforms, and anseriforms share an ancestral level of endocast doming that remained close to those of some non-volant Maniraptoriformes, such as *Halzskaraptor*, and their early volant relatives including *Archaeopteryx*. This larger phylogenetic range thus includes the endocast size and degree of endocast doming corresponding to the first dinosaurian “flight-adapted” brain. Endocast doming was found to resolve the developmental strategies associated with the evolution from non-maniraptoriform dinosaurs to Avialae and the subsequent evolutionary pathway towards and into neoavian birds. Nevertheless, strong group overlap and limited phylogenetic resolution at the origin and during the early evolutionary history of Avialae prevents identification of discrete conditions resolvable in lateral whole-endocast shape that either enabled volancy or unambiguously indicate volancy. Volancy originated in a dinosaurian group characterised by reduced body sizes relative to their ancestral condition that achieved sufficient cognitive performance to permit aerial locomotion. However, this locomotory innovation did not preserve a characteristic morphological hallmark on the endocranium, rendering the recognition of (early) dinosaurian volancy through whole-endocast volume^1^ and geometry (this contribution) impossible on presently known material.

The whole-endocast geometries of volant palaeognaths, galliforms, and anseriforms quantified in this study have deep evolutionary histories, as corroborated by their general proportions shared with some Maniraptoriformes, including *Archaeopteryx*. Agreement with the endocast shapes of the Paleocene volant palaeognath *Lithornis* and the Eocene anseriform *Presbyornis* furthermore indicate that these endocranial shapes are conservative relicts that do not represent (relatively) recent convergences. Among modern birds (Aves), volant palaeognaths, galliforms, and anseriforms are therefore concluded to preserve brain geometries most closely resembling that of the avian ancestor, which renders them most appropriate for reconstructing early avian cerebral developments.

The large brain shape diversity of Neoaves is mostly the product of complex and progressive niche partitioning that started during the early Paleogene neoavian diversification phase^61^ and set the stage for the exploitation of a broad ecological range occupied by present-day birds. This finding renders the explosive Paleogene avian niche expansion predominantly a neoavian affair, and opens up opportunities for studying endocranial shape variation in direct relation to avian ecological strategies. Nevertheless, it also challenges the employment of neoavian brain shape variation in reconstructing the early “flight-adapted” dinosaurian brain shape. Neoavian brain shape diversity reflects the influence of several successive developments resulting in a highly apomorphic brain shape range that has advanced beyond and thereby likely obscured informative morphological properties associated with the onset of dinosaurian volancy.

Following convergent progenetic developments associated with size reduction in the ancestral and early evolutionary histories of both birds and pterosaurs, we infer that certain evolutionary thresholds were negotiated in small-bodied ornithodirans that enabled the exploitation of volant ecologies, partially (but crucially) through exaptation of already available brain capacity (Fig. 1). After sufficient cerebral performance was allocated to enable aerial excursions, the evolutionary radiations of pterosaurs and birds both saw a complete decoupling of endocast doming and body size from the ancestral architecture. Within Pterosauria, progenetic effects on endocranial shape are particularly recognisable in derived ecologies, although increased flexure and cerebral enlargement were already recorded in the most basal pterosaurs studied herein. The origin of increased brain doming in the earliest volant dinosaurs was not intimately associated with volancy but rather with enhanced cerebral processing enabling increasingly complex behaviour.

A secondary radiation within neoavian birds was recorded in an explosive diversification of endocranial shapes, which reflected the establishment of a broad ecological range with variable volant requirements. Although recent results propose that even *Archaeopteryx* may already have been capable of flight^30^, its brain was probably not yet capable of controlling the complex volancy seen in most modern birds. Enantiornithes were probably proficient flyers^71–73^, and the basal ornithurine *Cerebavis* (circa 122 Ma) already featured clear flight-related adaptations in the cranium^14^. However, the fossil record surrounding the origin of Neornithes^74^, as well as generally limited aerial performance across the basalmost extant neornithine clades, suggests that the last Mesozoic ancestors of modern birds were likely much less capable flyers. None of the volant Palaeognathae or Galloanseriformes are capable of particularly dexterous aerial locomotion, and we found that they universally share low cerebral doming levels. However, neoavian taxa that rely on aerobatics for survival, including aerial foraging and predation in falcons and bee-eaters, hunting in and evading predation by negotiating the three-dimensional landscapes of woodlands and the undergrowth by respectively owls and small passerines, and optimising the aerial approach before plunge diving by Sulidae, tend to share substantial cerebral doming. We propose that demands for increased aerial dexterity, as those associated with behavioural optimisation in, for example, aerial foraging, or those imposed by habitat constraints, such as when traversing densily populated floral landscapes, partially drove the convergent cerebral doming that occurred in several neornithine groups. Although the modern avian diversity renders a categorical test of these relations particularly challenging, this hypothesis is consistent with earlier findings that visual cues stimulated both avian cerebral changes and volant capacities.

Avian flight mode itself does not directly influence brain volume^16^, nor does phylogeny^75,76^. However, cerebral organisation does respond to certain ecological changes^77^ and behavioural modifications^78,79^, such as those related to migration^80^. The evolution of avian flight closely ties in with changes in eye size and position^81^, and visual acuity^82^. Changing foraging modes are reflected in diverging brain shapes^83^, which has been related to the influence of increasing visual requirements^81^. Although many non-neornithine Mesozoic Avialae may have been proficient flyers relative to early Neornithes, this close relation between the wulst and visual acuity in extant birds^84^ nevertheless suggests that they were not necessarily employing flight as their dominant foraging mode.

## Methods

A digital library of high-resolution tomographical data on crania of 15 adult lepidosaurs, an ontogenetic series including 42 specimens of *Crocodylus niloticus*, 72 additional adult crocodilian individuals, two adult pterosaurs, four non-avian dinosaurs, four specimens of *Archaeopteryx*, 190 adult specimens of Neornithes, and ontogenetic series of *Gallus gallus*, *Phasianus colchicus, Ficedula albicollis* and *Passer domesticus*, was created using synchrotron X-ray microtomography at beamlines BM05, ID17 and ID19 of the European Synchrotron Radiation Facility in Grenoble, France. Synchrotron X-ray microtomography was conducted using an optimised polychromatic beam with sufficient coherence to allow for the use of PPC-SRμCT. Three-dimensional volume reconstruction was conducted through filtered back-projection following a single distance phase retrieval protocol that relies on an assumption of homogeneity^85,86^. The synchrotron data set was supplemented with an additional *Crocodylus niloticus* cranium visualised with a medical tomograph at the Centre Hospitalier Universitaire in Grenoble, France, and crania of one specimen of *Paleosuchus palpebrosus* and 146 adult Neornithes visualised using laboratory tomographical techniques at the Institute of Physiology and the Institute for Clinical and Experimental Medicine in Prague, Czech Republic. Additional cranial scanning data of 45 lepidosaurs, two crocodilians, and five Neornithes were accessed through DigiMorph (www.digimorph.com) and processed in tandem with the data obtained in the scope of this study. Material and data provenance as well as repositories curating the digital data are listed in Extended Data Table 1. Virtual three-dimensional endocasts were extracted from the reconstructed crania by adopting cranial bones as delimitating masks in VGStudio MAX 2.2 (Volume Graphics, Heidelberg, Germany). Additional appropriate endocasts visualised in lateral view were sourced from literature (Extended Data Table 1) and processed alongside the scanning data.

The holotype of the newly described paravian *Halszkaraptor escuilliei* presents valuable three-dimensional insight into morphological conditions of a basal Dromaeosauridae^29^, including those of its cranial endocast. Although its skeletal integrity has been extensively demonstrated^29^, we care to offer additional demonstration of the non-avialan paravian identity of the *Halszkaraptor* cranium. To that effect, we here briefly recall the cranial features in the *H. escuilliei* holotype that ally the cranium with Paraves but exclude it from Avialae. The holotype of *H.escuillei* presents typical paravian characteristics, including a frontoparietal suture located at the level of the postorbital process of the frontal^87^, a short lacrimal, and a tooth row reaching posteriorly to the base of the preorbital bar^29^. The specimen also exhibits a suite of three dromaeosaurid cranial synapomorphies that unequivocally render the skull of *Halszkaraptor* an appropriate representative for the non-avialan eumaniraptoran condition of the cranial endocast. These are the enlarged paraquadrate fenestra, the shallow and elongate preorbital maxilla, and the subparallel trending ventral and alveolar margins of the dentary^29,88^. The *Halszkaraptor* cranium does recall some avialan conditions, such as the ratio between orbital and anteorbital length exceeding 1.2^89^ and the dorsal border of the anteorbital fossa being formed by the nasal^87^. Nevertheless, because the lacrimal does not contribute to delimitation of the anteorbital fossa^87^, the lateral border of the quadrate is not a straight shaft^87^, and the nasal is longer than the frontal^89^, the cranium of *H. escuillei* is not avialan, as was corroborated by phylogenetic analysis^29^.

For specimens sourced from literature, developmental stages used in this study (Extended Data Table 1) correspond to those reported in the respective studies. The holotype of *Bambiraptor feinbergi* was first presented as an adult specimen^55^ but has been suggested to represent a sub-adult at 70% body length by Paul^90^. In light of our finding that post-hatchling C/D in archosaurs approaches the adult values quite closely, we have adopted its C/D as representative for the adult condition. In order to avoid bias in the C/D ratios resulting from retrodeformation, only braincases that conservatively preserve the braincase geometry informing the C/D ratio were employed in C/D analyses. Similarly, only endocasts obtained from well-preserved and representative specimens were sourced from literature for inclusion in the presented analyses.

The endocast of the London specimen of *Archaeopteryx* had already been reconstructed twice from a single data set obtained through conventional tomographical techniques^1,6^. The newly created endocasts of two specimens of *Archaeopteryx* (Fig. 3), including the London Specimen, that were obtained through PPC-SRμCT allowed for a re-examination of the potential presence of a wulst; a morphological structure of which the presence has been proposed to represent an unambiguous synapomorphy shared by *Archaeopteryx* and neornithes^1^. The London Specimen (BMNH 37001)^11^ is a nearly complete specimen of which most of the braincase was freed from the limestone slab. The left part of the natural cranial endocast remains largely delimited by cranial bones but the right side is directly observable because the corresponding part of the osseous braincase remains in the counterslab. Of the braincase in the Munich Specimen (BSP 1999 I 50)^11^, only a right parietal can be readily recognised, but more material may be present *ex situ* on and within the limestone plates.

On all adult archosaurian endocasts, seven Type I landmarks were conservatively identified in lateral view at discrete morphological locations along the dorsal endocast contour between the anteriormost tip of the telencephalon and the dorsalmost point of the foramen magnum. Two-dimensional landmark digitisation was realised in lateral views. Three-dimensional landmark visualisation was considered tenuous, as dinosaurian endocasts typically do not accurately preserve three-dimensional shape throughout. The definition of Type I landmarks relies on recognition of homologuous borders between cerebral structures, which in turns depend on the cerebral representativity of the endocast. This agreement is generally much lower in crocodilian and dinosaurian specimens than in modern avian material, which renders direct comparison challenging. Landmarks were placed on the borders between or extreme limits of cerebral structures (Extended Data Fig. 3c): 1-Anteriormost tip of telencephalon, 2-Border between telencephalon and cerebellum, 3-Dorsalmost tip of foramen magnum, 4-Ventralmost tip of foramen magnum, 5-Dorsalmost limit of telencephalon, 6- External border of cerebellum, equidistant from landmarks 2 and 3, and 7-Ventralmost expansion of the medulla oblongata, defined as the longest distance between the ventral surface of the medulla oblongata and the line between landmark 4 and the ventral contact between the medulla and the optic tectum.

Semilandmarks were placed at equidistant intervals along the dorsal endocast contour between the anteriormost tip of the telencephalon and the dorsalmost point of the foramen magnum in all studied adult archosaurian endocasts (Extended Data Fig. 3d) to capture whole-endocast shape. All landmarking was conducted in TpSDig2. Landmark-based 2D geometric morphometrics (GM) was used to quantify endocast shape and shape variation using MorphoJ (version 1.06b)^91^ following Procrustes superimposition. Shape analysis of the adult archosaurian subset was conducted by principal component analysis (PCA) in MorphoJ to explore shape variation among endocrania of adult archosaurs.

PCA scores were transferred to PAST (version 3.20)^92^ for visualisation and analysis. The correlation between the PCA results of Type I and semilandmarks was statistically assessed through regression analysis. Based on assessment of the most representative reflection of whole-endocast variability across archosaurs, the optimal landmark set appropriately capturing dominant whole-endocast shape variation in the total diapsid set was identified. The recovered dominant whole-endocast shape variability was translated into a simplified yet statistically warranted geometrical ratio. This novel shape ratio was calculated for the entire data set and plotted against log(endocast length) to visualise the associated endocast size variation. Relations and underlying patterns were assessed, interpreted, and discussed in relation to the evolutionary establishment of discrete archosaurian groups in the context of dinosaurian volancy (Extended Data Fig. 1). The divergence between the ontogenetic trajectories of *Crocodylus niloticus* and *Gallus gallus* (Fig. 4) was statistically tested through shape space trajectory analysis^93^ in Microsoft Excel (Microsoft Corporation, Redmond, Washington, USA; see Extended Data Table 1). Absolute age of the specimens was included as the log-transformed age in days after oviposition (Extended Data Table 1, Fig. 4a). Relative developmental age normalises absolute age (after oviposition) to homologuous developmental stages relative to hatchling (1) and the establishment of sexual maturity (2). As such, values between 0 and 1 reflect *in ovo* developmental stages, whereas values between 1 and 2 indicate hatchling, juvenile, and subadult to adult development (Extended Data Table 1, Fig. 4b).

Statistical tests for the relations between doming ratio and biological indices were conducted in the R environment (Rstudio 1.2.1355). Normality of body mass and alert cranial angle (i.e. head posture) was assessed using Shapiro-Wilk testing. As at least one of the variables was found to disagree with the assumption of normality, Kendall rank correlation tests were performed. Towards comparing doming ratio and encephalisation quotient, the dataset was first subjected to a Bartlett test to assess homoscedasticity of the dataset. The correlation between doming ratio and encephalisation quotient was subsequently tested independently for the different groups with the Kendall rank correlation test. The Kendall rank correlation test was selected over the Spearman correlation test because of its lower error sensitivity and the higher accuracy of its p-value. Results with p-value > 0.05 were considered as non-significant.

Towards defining birds, we follow Gauthier^94^ in recognising the synonymy of Neornithes and Aves. In this definition, only the last common ancestor of modern birds and all of its descendants account for the complete avian diversity. All dinosaurs with feathered wings used for flapping flight are placed within Avialae^94^, which renders Avialae virtually synonymous with the younger but now abandoned definition of Aves that encompasses the last of common ancestor of *Archaeopteryx* and *Passer domesticus*, and all of its descendants^95^.

## Supplementary information

Supplementary Dataset 2

Supplementary Dataset 1

Supplementary Dataset 3

## Data Availability

Data are accessible through the ESRF Paleontological Database (http://paleo.esrf.fr) and Morphobank (http://morphobank.org).
