# Supplementary material for: Multiphase progenetic development shaped the brain of flying archosaurs
Source: Sci Rep. 2019 Jul 25;9:10807. doi: 10.1038/s41598-019-46959-2 (PMC6658547; doi:10.1038/s41598-019-46959-2)
Supplement: Supplementary file 1 — Supplementary Dataset 2 [file 41598_2019_46959_MOESM1_ESM.pdf]

Multiphase progenetic development shaped the brain of flying archosaurs

Vincent Beyrand, Dennis F. A. E. Voeten, Stanislav Bureš, Vincent Fernandez, Jiří Janáček, Daniel Jirák, Oliver Rauhut, and Paul Tafforeau

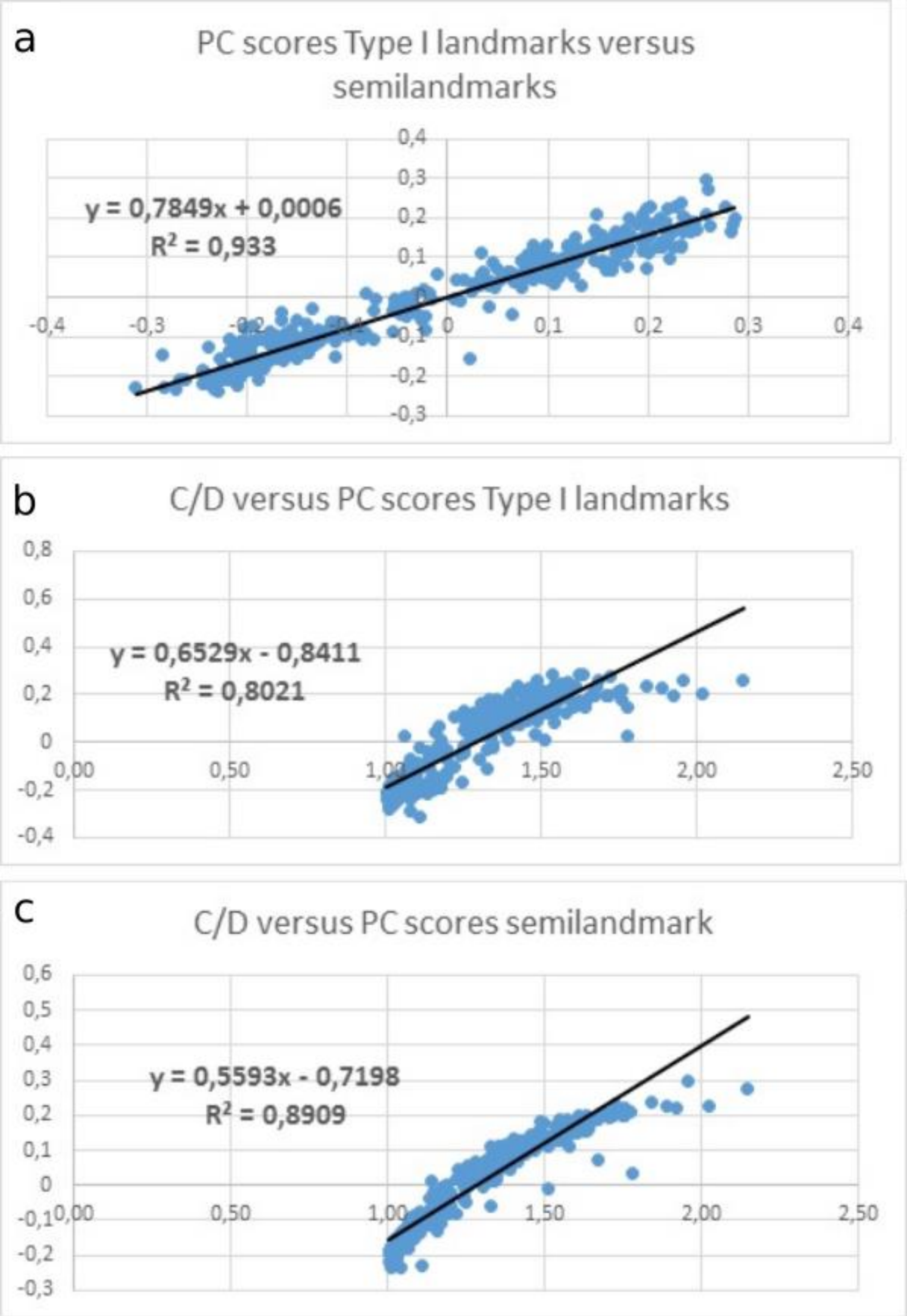

a
PC scores Type I landmarks versus semilandmarks
y = 0,7849x + 0,0006
R² = 0,933
-0,4
-0,3
-0,2
-0,1
0
0,1
0,2
0,3
0,4
b
C/D versus PC scores Type I landmarks
y = 0,6529x - 0,8411
R² = 0,8021
0,8
0,6
0,4
0,2
0
-0,2
-0,4
0,00
0,50
1,00
1,50
2,00
2,50
c
C/D versus PC scores semilandmark
y = 0,5593x - 0,7198
R² = 0,8909
0,6
0,5
0,4
0,3
0,2
0,1
0
-0,1
-0,2
-0,3
0,00
0,50
1,00
1,50
2,00
2,50

Extended Data Figure 1. Correlation plots between PC scores of Type I landmarks, semi-landmarks and endocranial doming (C/D). Correlation of PC Type I landmarks scores versus PC semi-landmark scores (a), C/D ratio versus PC Type I landmarks scores (b), C/D ratio versus PC semi-landmark scores (c).

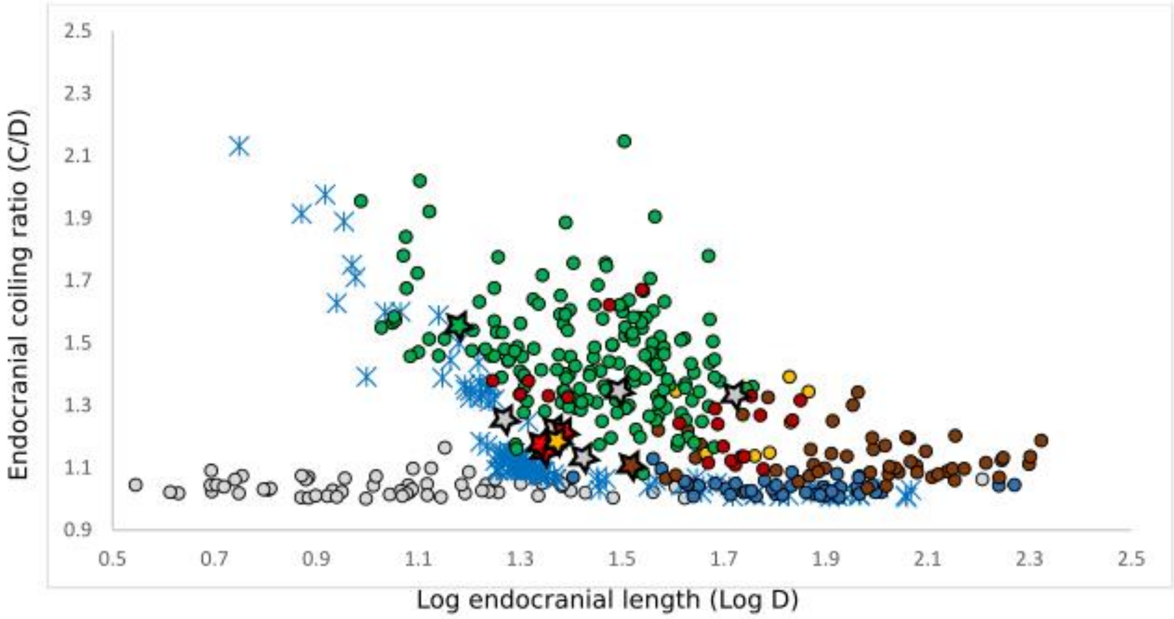

Extended Data Figure 2. Bivariate plot of log-transformed endocast length (Log D) versus endocranial doming (C/D) in all tested sauropsids. Small colour-coded circles represent phylogenetic groups: Lepidosauria (grey), crocodilians (blue), non-maniraptoriform dinosaurs (brown), Maniraptoriformes (yellow), Paleognathae (red), and Neognathae (green). Colour-coded stars represent key fossil endocasts of Pterosauria (grey), *Cerebavis cenomanica* (green), *Archaeopteryx lithographica* (red), *Halszkaraptor escuillei* (yellow), and *Heterodontosaurus tucki* (brown).

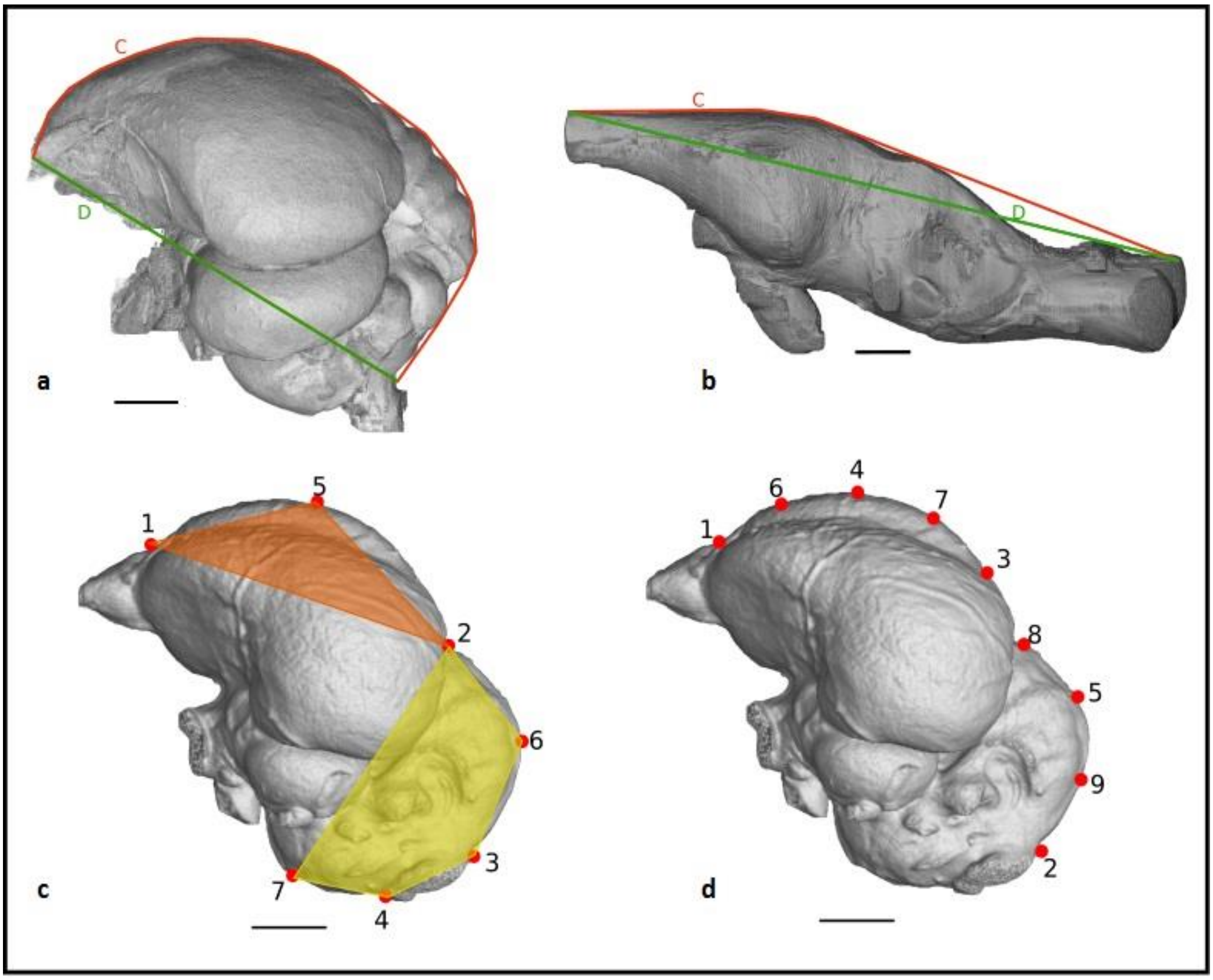

Extended Data Figure 3. Exemplified measurements of endocranial doming. Illustrated measurements for a) *Ficedula albicollis*, and b) *Crocodylus niloticus*. Green line captures endocast length between the anteriormost tip of the telencephalon and the opisthion ("D"). Red line depicts the length between the same points measured as a convex hull along the dorsal surface of the endocast ("C"). Positioning of Type I (c) and semilandmarks (d). Type I landmarks envelope telencephalic shape (orange area) and rhomboncephalic shape (yellow area). Scale bar length: a) 1.5 mm, b) 10 mm, c) and d) 5mm.

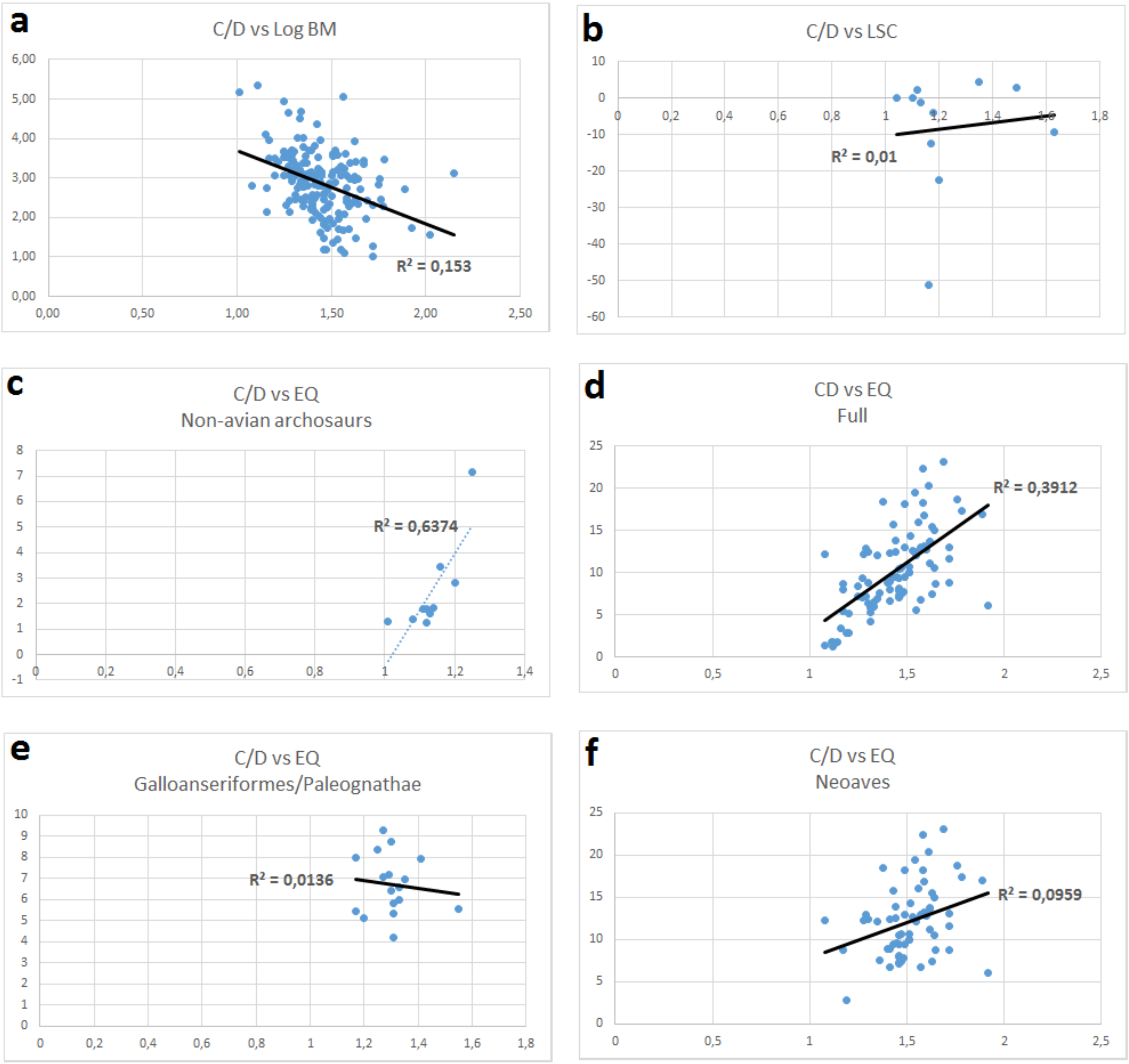

Extended Data Figure 4. Correlation plots between endocranial doming and biological indices. Correlation of C/D ratio versus log-transformed body mass (a), C/D ratio versus alert cranial angle[56] (b), C/D ratio versus encephalisation quotient for non-avian archosaurs (c), full archosaurian dataset (d), Galloanseriformes and Paleognathae (e) and Neoaves (f).

Extended Data Table 1

Abbreviations:

CT: Characterised using conventional X-ray Computed Tomography; ESRF: Characterised using synchrotron radiation based computed tomography; ESRF collection: Curated at the European Synchrotron Radiation Facility; LFAC: La Ferme aux Crocodiles, Pierrelattes, France; TL: Thierry Loeb, Echirolles, France; ENS: Ecole Normale Supérieure, Lyon, France; MHN Grenoble: Curated at Museum d'Histoire Naturelle, Grenoble, France; MNHN: Curated at Museum National d'Histoire Naturelle, Paris, France; CCEC: Centre de Conservation et d'Etude des Collections, Lyon, France; Peaugres: Safari de Peaugres, Peaugres, France.

Extended Data Table 2. Statistics of F-Test for Equality of Two Variances and t-test for Two Samples Assuming Unequal Variances comparing the ontogenetic trajectories of *Crocodylus niloticus* and *Gallus gallus*.

F-Test for Equality of Two Variances

	Variable 1	*Variable 2*
Mean	1,55557807	1,32548981
Variance	0,10694632	0,07108515
Observations	13	46
df	12	45
F	1,50448181	
P(F<=f) one-tail	0,15808312	
F Critical one-tail	1,97449795	

t-test: Two-Sample Assuming Unequal Variances

	Variable 1	*Variable 2*
Mean	1,55557807	1,32548981
Variance	0,10694632	0,07108515
Observations	13	46
Pooled Variance	0,07863487	
Hypothesised Mean Difference	0	
df	57	
t Stat	2,61223033	
P(T<=t) one-tail	0,00573997	
t Critical one-tail	1,67202889	
P(T<=t) two-tail	0,01147995	
t Critical two-tail	2,00246546	

Extended Data Table 3

Abbreviations:

BM: Body Mass for adult specimens[42,53,54] and crocodilian ontogenetic series[55]; LSC: Lateral Semicircular Canal orientation used for assessing head posture[56]; EQ: Encephalisation Quotient[57];

Extended Data Table 4. Statistics for correlation between biological indices and C/D ratio.

	p-value	
Shapiro test for CD	0.0005713	
Shapiro test for body mass	0.07662	Kendall tau
Correlation for CD vs body mass	5.757e-08	-0.2787634

	p-value	
Shapiro test for CD	0.02488	
Shapiro test for LSC angle	0.001813	Kendall tau
Correlation for CD vs LSC angle	0.8793	-0.05454545

Extended Data Table 5. Statistics for correlation between encephalisation quotient and C/D ratio.

	p-value
Bartlett Test	0.001815

Groups	p-value	Kendall tau
Non-avian archosaurs	0.007058	0.6741999
Full dataset	2.968e-11	0.4825782
Paleognathae/Galloanserae	0.534	-0.1132471
Neoaves	0.005265	0.2430949

Supplementary References

1. Lautenschlager, S. & Butler, R.J. Neural and endocranial anatomy of Triassic phytosaurian reptiles and convergence with fossil and modern crocodylians. *PeerJ*. **4**, e2251, 10.7717/peerj.2251 (2016).
2. Hopson, J.A. Paleoneurology in *Biology of the Reptilia (Neurology).* (ed. Gans, C., Northcutt, R.G. & Ulinski, P.) **9**, 39-146 (Academic Press New York, 1979).
3. von Baczko, M.B. & Desojo, J.B. 2016. Cranial anatomy and paleoneurology of the archosaur *Riojasuchus tenuisceps* from the Los Colorados Formation, La Rioja, Argentina. *PLOS ONE* **11**, e0148575, 10.1371/journal.pone.0148575 (2016).
4. George, L.D. & Holliday, C.M. Trigeminal nerve morphology in *Alligator mississipiensis* and its significance for crocodyliform facial sensation and evolution. *Anat. Rec.* **296**, 670-690 (2013).
5. Dufeau, D.L. & Witmer, L.M. Ontogeny of the middle-ear air-sinus system in *Alligator mississipiensis* (Archosauria : Crocodylia). *PLOS ONE*, e0137060, 10.1371/journal.pone.0137060 (2015).
6. Pierce, S.E., Williams, M. & Benson, R.B.J. Virtual reconstruction of the brain and sinuses of the Early Jurassic marine crocodylomorph *Pelagosaurus typus* (Thalattosuchia). *PeerJ Preprints*, e2539v1, 107717/peerj.3225 (2016).
7. Cordoniú, L., Carabajal, A.P., Pol, D., Unwin, D. & Rauhut, O. A Jurassic pterosaur from Patagonia and the origin of the pterodactyloid neurocranium. *PeerJ* **4**, e2311, 10.7717/peerj.2311 (2016).
8. Witmer L.M. Inside the oldest bird brain. Nature **430**, 619-620 (2004).
9. Witmer, L.M. & Ridgely, R.C. Structure of the brain cavity and inner ear of the centrosaurine ceratopsid dinosaur *Pachyrhinosaurus* based on CT scanning and 3D visualization in *A new horned dinosaur from an Upper Cretaceous bone bed in Alberta* (ed. Currie, P.J.) 117-144 (National Research Council Research Press, Ottawa, 2008).
10. Zhou, C.F., Gao, K.Q., Fox, R.C. & Du, X.K. Endocranial morphology of psittacosaurs (Dinosauria: Ceratopsia) based on CT scans of new fossils from the Lower Cretaceous, China. *Palaeoworld* **16**, 285-293 (2007).
11. Lauters, P., Vercauteren, M., Bolotsky, Y.L. & Godefroit, P. Cranial endocast of the lambeosaurine hadrosaurid *Amurosaurus riabinini* from the Amur Region, Russia. *PLOS ONE* **8**, e78899, 10.1371/journal.pone.0078899 (2013).

12. Evans, D.C., Ridgely, R. & Witmer, L.W. Endocranial anatomy of lambeosaurine hadrosaurids (Dinosauria : Ornithischia) : A sensorineural perpspective on cranial crest function. *Anat. Rec.* **292,** 1315-1337 (2009).
13. Hurlburt, G.R. Were dinosaurs cold-or warm-blooded?: An exercise in scientific inference. *Tested studies for laboratory teaching* **15**, 181-214 (1994).
14. Farke, A.A., Chok, D.J., Herrero, A., Scolieri, B. & Werning, S. Ontogeny in the tube-crested dinosaur *Parasaurolophus* (Hadrosauridae) and heterochrony in hadrosaurids. *PeerJ.* **1**, e182, 10.7717/peerj.182 (2013).
15. Thomas, D.A. The cranial anatomy of *Tenontosaurus tilletti* Ostrom, 1970 (Dinosauria, Ornithopoda*). Palaeontologica Electronica.***18.2**, 1-99, 10.2687/450 (2015).
16. Cruzado-Caballero, P., Fortuny, J., Llacer, S. & Canudo, J.I. *Arenysaurus ardevoli*, first paleoneuroanatomical description of a European hadrosaurid. *PeerJ Preprints*, e590v1, 10.7287/peerj.preprints.590v1 (2014).
17. Lautenschlager, S. & Hübner, T. Ontogenetic trajectories in the ornithischian endocranium. *J. Evol. Biol.* **26**, 2044-2050 (2013).
18. Brasier, M.D. et *al*. Remarkable preservation of brain tissues in an Early Cretaceous iguanodontian dinosaur. *Geol. Soc. London Spec. Publ.* **448,** 1-16 (2016).
19. Miyashita, T., Arbour, V.M., Witmer, L.M. & Currie, P.J. The internal cranial morphology of an armoured dinosaur *Euoplocephalus* corroborated by X-ray computed tomographic reconstruction. *J. Anat.* **219**, 661-675 (2011).
20. Leahey, L.G., Molnar, R.E., Carpenter, K., Witmer, L.M. & Salisbury, S.W. Cranial osteology of the ankylosaurian dinosaur formerly known as *Minmi sp.* (Ornithischia: Thyreophora) from the Lower Cretaceous Allaru Mudstone of Richmond, Queensland, Australia. *PeerJ*. **3**, e1475, 10.7717/peerj.1475 (2015).
21. Carabajal, A.P., Lee, Y.N. & Jacobs, L.L. Endocranial morphology of the primitive nodosaurid dinosaur *Pawpawsaurus campbelli* from the Early Cretaceous of North America. *PLOS ONE* **11**, e 0150845, 10.1371/journal.pone.0150845 (2016).
22. Zheng, Z. Cranial anatomy of *Shunosaurus* and *Camarasaurus* (Dinosauria : Sauropoda) and the phylogeny of the Sauropoda. 208 pp.(Texas Tech University, 1996).
23. Carabajal, A.P., Carballido, J.L. & Currie, P.J. Braincase, neuroanatomy, and neck posture of *Amargasaurus cazaui* (Sauropoda, Dicraeosauridae) and its implications for understanding head posture in sauropods. *J. Vertebr. Paleontol.* **34**, 870-882 (2014).

24. Witmer, L.M. & Ridgely, R.C. New insights into the brain, braincase and ear region of Tyrannosaurs (Dinosauria, Theropoda), with implications for sensory organisation and behavior. *Anat. Rec.* **292**, 1266-1296 (2009).
25. Sereno, P.C. et *al*. Structural extremes in a Cretaceous Dinosaur. *PLOS ONE* **2**, e1230, 10.1371/journal.pone.0001230 (2007).
26. Carabajal, A.P. Neuroanatomy of titanosaurid dinosaurs from the Upper Cretaceous of Patagonia, with comments on endocranial variability within Sauropoda. *Anatomic. Rec.* **295**, 2141-2156 (2012).
27. Knoll, F., Ridgely, R.C., Ortega, F., Sanz, J.L. & Witmer, L.M. Neurocranial osteology and neuroanatomy of a Late Cretaceous titanosaurian sauropod from Spain (*Ampelosaurus sp.*). *PLOS ONE*. **8**, p.e54991, 10.1371/journal.pone.0054991 (2013).
28. Knoll, F., Witmer, L.M., Ortega, F., Ridgely, R.C. & Schwarz-Wings, D. The braincase of the basal sauropod dinosaur *Spinophorosaurus* and 3D reconstructions of the cranial endocast and inner ear. *PLOS ONE*. **7**, e30060, 10.1371/journal.pone.0030060 (2012).
29. Carabajal, A.P. & Succar, C. The endocranial morphology and inner ear of the abelisaurid theropod *Aucasaurus garridoi*. *Acta Palaeontol. Pol.* **60**, 141-144, 10.4202/app.2013.0037 (2015).
30. Sampson, S.D. & Witmer L.M. Craniofacial anatomy of *Majungasaurus crenatissimus* (Theropoda: Abelisauridae) from the Late Cretaceous of Madagascar. *J. Vertebr. Paleontol.* **27**, 32-102 (2007).
31. Franzosa J.W. Evolution of the Brain in Theropoda (Dinosauria). 357pp (University of Texas, 2004).
32. Larsson, H.C.E. Endocranial anatomy of *Carcharodontosaurus saharicus* (Theropoda: Allosauroidea) and its implications for theropod brain evolution in *Mesozoic Vertebrate Life* (ed. Tanke, D.H. & Carpenter K.) 19-33 (Indiana University Press, 2001).
33. Carabajal, A.P. & Canale, J.I. Cranial endocast of the carcharodontosaurid theropod *Giganotosaurus carolinii* Coria & Salgado,1995. *Neues Jahrb. Geol. Paläontol.* **258**, 249-256 (2010).
34. Sanders, R.K. & Smith, D.K. The endocranium of the theropod dinosaur *Ceratosaurus* studied with computer tomography. *Acta Palaeontol. Pol.* **50**, 601-616 (2005).
35. Xing, L.et *al.* Braincase anatomy of the basal theropod *Sinosaurus* from the Early Jurassic of China. *Acta Geol. Sin.* **88**, 1653-1664 (2014).

36. Carabajal, A.P. & Currie, P.J. New information on the braincase of *Sinraptor dongi* (Theropoda, Allosauroidea): ethmoidal region, endocranial anatomy and pneumaticity. *Vert. PalAs.* **50**, 85-101 (2012).
37. Bever, G.S., Brusatte, S.L., Balanoff, A.M. & Norell, M.A. Variation, variability and the origin of the avian endocranium : insights from *Alioramus altai*. *PLOS ONE* **6**, e23393, 10.1371/journal.pone.0023393 (2011).
38. Saveliev, S.V. & Alifanov, V.R. A new study of the brain of the predatory dinosaur, *Tarbosaurus bataar* (Theropoda, Tyrannosauridae). *Paleontol. J.* **41**, 281-289 (2007).
39. Lautenschlager, S., Rayfield, E.J., Altangerel, P., Zanno, L.E. & Witmer, L.M. The endocranial anatomy of Therizinosauria and its implications for sensory and cognitive function. *PLOS ONE* **7**, e52289, 10.1371/journal.pone.0052289 (2012).
40. Cuff, A.R. & Rayfield, E.J. Retrodeformation and muscular reconstruction of ornithomimosaurian dinosaur crania. *PeerJ,* e1093, 10.7717/peerj.1093 (2015).
41. Tahara, R. & Larsson, C.E. Cranial pneumatic anatomy of *Ornithomimus edmontonicus* (Ornithomimidae: Theropoda). *J. Vertebr. Paleontol.* **31**, 127-143 2011).
42. Balanoff, A.M., Bever, G.S., Rowe, T.B. & Norell M.A. Evolutionary origins of the avian brain. *Nature* **501** (7465), 93-96 (2013).
43. Balanoff, A.M., Xing, X., Kobayashi, Y., Matsufune, Y. & Norell M.A. Cranial osteology of the Theropod dinosaur *Incisivosaurus gauthieri* (Theropoda: Oviraptosauria). *Am. Mus. Novit.* **3651** (2009).
44. Burnham, D.A. New information on *Bambiraptor feinbergi* (Theropoda: Dromaeosauridae) from the Late Cretaceous of Montana in *The feathered dragons* (ed. Currie, P.J., Koppelhus, E., Shugar, M.A. & Wright, J.). 67-111 (Indiana University Press, 2004).
45. Alonso, P.D., Milner, A.C., Ketcham, R.A., Cookson, M.J. & Rowe T.B. The avian nature of the brain and inner ear of *Archaeopteryx*. *Nature* **430** (7000), 666-669 (2004).
46. Walsh, S.A., Milner, A.C. & Bourdon, E. A reappraisal of *Cerebavis cenomanica* (Aves, Ornithurae) from Melovatka, Russia. *J. Anat.* **229**, 1-13 (2016).
47. Ashwell, K.W.S. & Scofield, R.P. Big birds and their brains: Paleoneurology of the New Zealand Moa. *Brain Behav. Evol.* **71**, 151-166 (2008).
48. Zelenitsky, D.K., Therrien, F., Ridgely R.C., McGee, A.R. & Witmer, L.M. Evolution of olfaction in non-avian theropod dinosaurs and birds. *Proc. R. Soc. B* **278**, 3625-3634 (2011).

49. Picasso, M.B.J., Tambussi C.P. & Degrange F.J. Virtual reconstructions of the endocranial cavity of *Rhea americana* (Aves, Palaeognathae): postnatal anatomical changes. *Brain Behav. Evol.* **76**, 176-184 (2010).
50. Corfield, J.R., Wild, J.M., Hauber, M.E., Parsons, S. & Kubke, F.B. Evolution of brain size in the palaeognath lineage, with an emphasis on New Zealand ratites. *Brain Behav. Evol.* **71**, 87-99 (2008).
51. Gold, M.E.L., Bourdon, E. & Norell, M.A. The first endocast of the extinct dodo (*Raphus cucculatus*) and an anatomical comparison amongst close relatives (Aves, Columbiformes). *Zool. J. Linn. Soc.* **177**, 950-963 (2016).
52. Romick C.A. Ontogeny of the brain endocasts of ostriches (Aves: *Struthio camelus*) with implications for interpreting extinct dinosaur endocasts. (Ohio University, 2013).
53. .Dunning Jr, J.B. CRC Handbook of avian body masses. (CRC Press, 2007).
54. Minias, P. & Podlaszczuk, P. Longevity is associated with relative brain size in birds. *Ecol. Evol*. **7** (10), 3558-3566 (2017).
55. Peterka, M. et *al.* Prenatal development of *Crocodylus niloticus niloticus* Laurenti, 1768. *J. Exp. Zool. B Mol. Dev. Evol*. **314** (5), 353-368 (2010).
56. Marugán-Lobón, J., Chiappe, L.M. & Farke, A.A. The variability of inner ear orientation in saurischian dinosaurs: testing the use of semicircular canals as a reference system for comparative anatomy. *PeerJ* **1**, e124.
57. Jerison, H.J. Evolution of the brain and intelligence. 465 pp. (New York: Academic Press, 1973).
